# Supplementary material for: A prediction method of fire frequency: Based on the optimization of SARIMA model
Source: PLoS One. 2021 Aug 9;16(8):e0255857. doi: 10.1371/journal.pone.0255857 (PMC8352067; doi:10.1371/journal.pone.0255857)
Supplement: S1 Data — (DOCX) [file pone.0255857.s001.docx]

**Appendix 1**

**Tabel 1 Monthly change of fire in China from 2003 to 2017**

| Year | Month | Fire frequency |
| --- | --- | --- |
| 2003 | 1 | 26788 |
|  | 2 | 30155 |
|  | 3 | 23131 |
|  | 4 | 23448 |
|  | 5 | 20240 |
|  | 6 | 18707 |
|  | 7 | 17777 |
|  | 8 | 16359 |
|  | 9 | 14502 |
|  | 10 | 17758 |
|  | 11 | 20226 |
|  | 12 | 24841 |
| 2004 | 1 | 37257 |
|  | 2 | 29684 |
|  | 3 | 26330 |
|  | 4 | 24056 |
|  | 5 | 20142 |
|  | 6 | 19644 |
|  | 7 | 15422 |
|  | 8 | 13979 |
|  | 9 | 12854 |
|  | 10 | 17983 |
|  | 11 | 17358 |
|  | 12 | 18095 |
| 2005 | 1 | 23253 |
|  | 2 | 28219 |
|  | 3 | 24855 |
|  | 4 | 23395 |
|  | 5 | 18285 |
|  | 6 | 17979 |
|  | 7 | 14346 |
|  | 8 | 13142 |
|  | 9 | 13378 |
|  | 10 | 15928 |
|  | 11 | 19573 |
|  | 12 | 23588 |
| 2006 | 1 | 28671 |
|  | 2 | 24405 |
|  | 3 | 24927 |
|  | 4 | 22420 |
|  | 5 | 19783 |
|  | 6 | 17949 |
|  | 7 | 14691 |
|  | 8 | 16335 |
|  | 9 | 13713 |
|  | 10 | 14978 |
|  | 11 | 16797 |
|  | 12 | 17212 |
| 2007 | 1 | 18611 |
|  | 2 | 24906 |
|  | 3 | 13531 |
|  | 4 | 14154 |
|  | 5 | 15642 |
|  | 6 | 12655 |
|  | 7 | 9912 |
|  | 8 | 9167 |
|  | 9 | 8801 |
|  | 10 | 11477 |
|  | 11 | 12033 |
|  | 12 | 12632 |
| 2008 | 1 | 14443 |
|  | 2 | 21700 |
|  | 3 | 14138 |
|  | 4 | 10899 |
|  | 5 | 10827 |
|  | 6 | 8989 |
|  | 7 | 7991 |
|  | 8 | 7507 |
|  | 9 | 7381 |
|  | 10 | 8335 |
|  | 11 | 10607 |
|  | 12 | 14018 |
| 2009 | 1 | 20554 |
|  | 2 | 11495 |
|  | 3 | 10906 |
|  | 4 | 10931 |
|  | 5 | 10641 |
|  | 6 | 9287 |
|  | 7 | 8020 |
|  | 8 | 7594 |
|  | 9 | 7425 |
|  | 10 | 10095 |
|  | 11 | 10133 |
|  | 12 | 12301 |
| 2010 | 1 | 13734 |
|  | 2 | 18009 |
|  | 3 | 11682 |
|  | 4 | 10707 |
|  | 5 | 9649 |
|  | 6 | 9017 |
|  | 7 | 8225 |
|  | 8 | 8608 |
|  | 9 | 7520 |
|  | 10 | 9382 |
|  | 11 | 11569 |
|  | 12 | 14395 |
| 2011 | 1 | 16862 |
|  | 2 | 19565 |
|  | 3 | 14036 |
|  | 4 | 14494 |
|  | 5 | 10764 |
|  | 6 | 8822 |
|  | 7 | 8320 |
|  | 8 | 8289 |
|  | 9 | 7752 |
|  | 10 | 7403 |
|  | 11 | 4667 |
|  | 12 | 4443 |
| 2012 | 1 | 14315 |
|  | 2 | 11497 |
|  | 3 | 12036 |
|  | 4 | 14566 |
|  | 5 | 12642 |
|  | 6 | 13111 |
|  | 7 | 10857 |
|  | 8 | 10587 |
|  | 9 | 11006 |
|  | 10 | 14249 |
|  | 11 | 12963 |
|  | 12 | 14328 |
| 2013 | 1 | 34325 |
|  | 2 | 36271 |
|  | 3 | 34961 |
|  | 4 | 33593 |
|  | 5 | 31302 |
|  | 6 | 27189 |
|  | 7 | 28851 |
|  | 8 | 29670 |
|  | 9 | 24799 |
|  | 10 | 33385 |
|  | 11 | 32411 |
|  | 12 | 40064 |
| 2014 | 1 | 67668 |
|  | 2 | 41501 |
|  | 3 | 39818 |
|  | 4 | 36151 |
|  | 5 | 32811 |
|  | 6 | 26689 |
|  | 7 | 25426 |
|  | 8 | 21569 |
|  | 9 | 19653 |
|  | 10 | 27263 |
|  | 11 | 24207 |
|  | 12 | 32296 |
| 2015 | 1 | 33134 |
|  | 2 | 41577 |
|  | 3 | 37267 |
|  | 4 | 34652 |
|  | 5 | 30696 |
|  | 6 | 28457 |
|  | 7 | 24546 |
|  | 8 | 24011 |
|  | 9 | 21070 |
|  | 10 | 26238 |
|  | 11 | 21460 |
|  | 12 | 23593 |
| 2016 | 1 | 33411 |
|  | 2 | 44972 |
|  | 3 | 32631 |
|  | 4 | 29188 |
|  | 5 | 25730 |
|  | 6 | 23756 |
|  | 7 | 24856 |
|  | 8 | 22385 |
|  | 9 | 20688 |
|  | 10 | 20647 |
|  | 11 | 21839 |
|  | 12 | 23533 |
| 2017 | 1 | 34858 |
|  | 2 | 28134 |
|  | 3 | 27386 |
|  | 4 | 29646 |
|  | 5 | 25448 |
|  | 6 | 20648 |
|  | 7 | 21530 |
|  | 8 | 17423 |
|  | 9 | 16525 |
|  | 10 | 16867 |
|  | 11 | 19627 |
|  | 12 | 23375 |

**Appendix 2**

**Tabel 1 Characteristics of fire distribution by hour from 2015-2017**

| **Year** | **time** | **Number of fire** | **Death toll** |
| --- | --- | --- | --- |
| **2017** | 0-2 | 15792 | 175 |
|  | 2-4 | 11991 | 207 |
|  | 4-6 | 10578 | 221 |
|  | 6-8 | 13183 | 82 |
|  | 8-10 | 22103 | 85 |
|  | 10-12 | 30324 | 88 |
|  | 12-14 | 31442 | 58 |
|  | 14-16 | 34410 | 78 |
|  | 16-18 | 32391 | 87 |
|  | 18-20 | 32355 | 92 |
|  | 20-22 | 26771 | 88 |
|  | 22-24 | 20127 | 129 |
| **2016** | 0-2 | 17777 | 212 |
|  | 2-4 | 13689 | 217 |
|  | 4-6 | 11922 | 186 |
|  | 6-8 | 15470 | 126 |
|  | 8-10 | 25226 | 115 |
|  | 10-12 | 34750 | 102 |
|  | 12-14 | 35888 | 77 |
|  | 14-16 | 39080 | 86 |
|  | 16-18 | 37134 | 104 |
|  | 18-20 | 37927 | 89 |
|  | 20-22 | 31707 | 106 |
|  | 22-24 | 23066 | 171 |
| **2015** | 0-2 | 19341 | 241 |
|  | 2-4 | 14701 | 280 |
|  | 4-6 | 12399 | 194 |
|  | 6-8 | 15892 | 145 |
|  | 8-10 | 27059 | 112 |
|  | 10-12 | 37078 | 137 |
|  | 12-14 | 38435 | 131 |
|  | 14-16 | 41900 | 97 |
|  | 16-18 | 39718 | 108 |
|  | 18-20 | 40805 | 118 |
|  | 20-22 | 34173 | 107 |
|  | 22-24 | 25200 | 229 |
