# Supplementary material for: A prediction method of fire frequency: Based on the optimization of SARIMA model
Source: PLoS One. 2021 Aug 9;16(8):e0255857. doi: 10.1371/journal.pone.0255857 (PMC8352067; doi:10.1371/journal.pone.0255857)
Supplement: Attachment — Submitted filename: Response to Reviewers.docx [file pone.0255857.s002.docx]

Dear Editor,

Thank you very much for giving us an opportunity to revise our manuscript. We appreciate the editor and reviewers very much for their constructive comments and suggestions on our manuscript entitled “A prediction method of fire frequency: Based on the optimization of SARIMA model” (ID: PONE-D-21-17208).

We have studied the journal requirements carefully. The main corrections are in the manuscript and the responds to the editor’s comments are as follows (**the replies are highlighted in blue**).

1. Please ensure that your manuscript meets PLOS ONE's style requirements, including those for file naming.

Response：**We have carefully read the style requirements of the given website and confirmed that the manuscript meets PLOS ONE's style requirements.**

2. Thank you for stating the following in the Acknowledgments Section of your manuscript:

"This work is funded by National Key R&D Program of China (No.

2018YFC0809700), the basic research business expenses of CUEB (No.

XRZ2020018)"

We note that you have provided funding information that is not currently declared in your Funding Statement. However, funding information should not appear in the Acknowledgments section or other areas of your manuscript. We will only publish funding information present in the Funding Statement section of the online submission form.

Please remove any funding-related text from the manuscript and let us know how you would like to update your Funding Statement. Currently, your Funding Statement reads as follows:

"YES - Specify the role(s) played."

Please include your amended statements within your cover letter; we will change the online submission form on your behalf.

Response：**We have made the changes as required.**

**"This work is funded by National Key R&D Program of China (No.**

**2018YFC0809700), the basic research funds of Capital University of Economics and Business (No.XRZ2020018)"**

**① The sources of funding: Nationanl Key R&D Program of China，**

**Number: 2018YFC0809700**

**Recipient: Yudong Zhang**

**The role the funders took in the study: The funders give guidance in the writing of the manuscript.**

**② The sources of funding: The basic research funds of Capital University of Economics and Business，**

**Number: XRZ2020018**

**Recipient: Shuqi Ma**

**The role the funders took in the study: The funders participated in study design, data collection and analysis, decision to publish, and preparation of the manuscript.**

3. In your Data Availability statement, you have not specified where the minimal data set underlying the results described in your manuscript can be found. PLOS defines a study's minimal data set as the underlying data used to reach the conclusions drawn in the manuscript and any additional data required to replicate the reported study findings in their entirety. All PLOS journals require that the minimal data set be made fully available.

Response： **The data set used in this paper is all from China Fire Service, which is a book kept in the National Library of China. There is no website to search these data. I saved the data in the file “Data” and submitted it to the submission system as an attachment.**

Kind regards.

Shuqi Ma

E-mail: shuqi_10@163.com

Corresponding author : Qianyi LIU, Yudong ZHANG

E-mail address: [lqy202103@163.com](mailto:lqy202103@163.com); zhangyudong@tsinghua.edu.cn

We have studied reviewers’ comments carefully. According to the reviewers’ detailed suggestions, we have made a careful revision on the original manuscript. All revised portions are marked in red in the revised manuscript which we would like to submit for your kind consideration.

Dear reviewers:

Thank you for your comments on our manuscript entitled " A prediction method of fire frequency: Based on the optimization of SARIMA model" (ID：PONE-D-21-17208). Those comments are very helpful for revising and improving our paper, as well as the important guiding significance to other research. We have studied the comments carefully and made corrections which we hope meet with approval. The main corrections are in the manuscript and the responds to the reviewers’ comments are as follows (the replies are highlighted in blue ).

Replies to the reviewers’ comments:

Reviewer #1:

1. Is the manuscript technically sound, and do the data support the conclusions?

Response：**I have made detailed amendments to the specific problems raised by the Reviewer.**

2. Has the statistical analysis been performed appropriately and rigorously?

Response：**Thank you for your approval.** **We also made some improvements in the manuscript.**

3. Have the authors made all data underlying the findings in their manuscript fully available?

Response： **Thank you for your approval. We also made some improvements in the manuscript.**

4. Is the manuscript presented in an intelligible fashion and written in standard English?

Response：**Thank you for your approval. We also made some improvements in the manuscript.**

5. Review Comments to the Author

1) There is an error in Table 2 with 23,7 万， it's Chinese unit.

Response：**This is my negligence, which has been corrected in the manuscript.**

2) The fire data in the paper includes forest fires and urban fires, but forest fires are mainly affected by climatic factors, and urban fires are mainly affected by human factors. Whether the data application is reasonable with this model？

Response：**We mainly focus the occurrence law and prediction of fire frequency in China in this paper, and the influencing factors of different types of fire are less considered in this paper. We will consider the relevant aspects when analyzing the influencing factors of fire in other papers.**

3) Please explain why the number of fires in January 2014 is obviously abnormal？

Response：**On the one hand, due to seasonal reasons, people use more electricity and fire in winter, and the weather is dry and the wind speed is high, so the fire frequency is relatively high in January or February of each year. On the other hand, according to statistics, the fire incidence is high during the Spring Festival, accounting for more than 30% of that month. January 2014 is new year's Eve, and the fire proportion during the Spring Festival of that year is as high as 43%, The famous Shangri La fire also happened this month, and other unknown factors caused more fires in that month.**

Reviewer #2:

1. Is the manuscript technically sound, and do the data support the conclusions?

Response：**I have made detailed amendments to the specific problems raised by the Reviewer.**

2. Has the statistical analysis been performed appropriately and rigorously?

Response：**I have made detailed amendments to the specific problems raised by the Reviewer.**

3. Have the authors made all data underlying the findings in their manuscript fully available?

Response：**I have made detailed amendments to the specific problems raised by the Reviewer.**

4. Is the manuscript presented in an intelligible fashion and written in standard English?

Response：**I have made detailed amendments to the specific problems raised by the Reviewer.**

5. Review Comments to the Author

1) The Introduction is difficult to follow and does not set the reader up to understand the research objectives and how they address a gap in knowledge. The grammar needs to be addressed, and entire concepts like what type of fire events are even the focus of the paper (natural or anthropogenic) are entirely lost.

Response： **I have made a detailed revision to the questions raised by the reviewer.**

2) Line 15: What fire department?

Response：**The Fire and Rescue Department Ministry of Emergency Management, and it's been corrected in the manuscript.**

3) Line 18: What kind of fire data, natural (e.g., forests, ecosystems) or anthropogenic (human infrastructure), because in the previous sentences the authors introduced natural and anthropogenic fire examples.

Response：**Include natural fire and anthropogenic fire data. It's been corrected in the manuscript by “In the past, descriptive statistical methods were often used in the fire situation analysis of natural fire and anthropological fire”.**

4) Line 19: fire frequency as it relates to what kind? (see previous comment

Response：**Include natural fire and anthropogenic fire data.**

5)Line 33: Define “ANN” and “SVM”

Response：**ANN is Artificial Neural Network. SVM is Support Vector Machine.** **It's been corrected in the manuscript.**

6)Line 36: “used”; should “Big” be capitalized?

Response：**This is my negligence, which has been corrected in the manuscript.**

7)Line 43: earlier than what?

Response：**Correct the sentence as follows: For example, the construction of fire information in European and American started earlier than other countries, and many cities in these countries have established highly perfect urban fire alarm systems** **which usually have comprehensive prevention and control capabilities such as fire data collection, summary, statistics, prediction and early warning.**

8)Line 44: fire alarm systems related to what? See Line 18 comment.

Response：**It's urban fire alarm systems.**

9)Line 46-48: Revise sentence structure

Response： **Moreover, the fire management center can respond in time, and make analysis and decision rapidly^[11]^.**

10)Line 55: edit stochastic

Response：**It has been corrected in the manuscript**

11)Line 59: in the early stage of what? Next sentence: what does “it” refer to?

Response：**In the early stage of this century.“it” refer to fire prediction.**

12) Line 62: Not appropriate use of the pronoun “it”

Response：**By analyzing the development and exploration process of fire risk prediction technology, we found that there are common problems such as model accuracy, data quality, factor selection and so on.**

13)Line 67: What is meant by “to realize”

Response：**In order** **to realize**

Methods:

14) What is the China Fire YearBook? What types of fire data are the authors referring to? The methods section presents the statistical model forms, but does not present the actual approach to evaluating the fire data.

Response：**After our confirmation, it should be "China Fire Services", which is a book compiled by the Fire Department of the Ministry of Public Security, including the annual national fire work and fire brigade construction, fire cases, national fire statistics, etc.**

15) Line 80: “In the 20th century” is likely not needed consider removing.

Response：**It has been deleted.**

16)Line 82: Revise for English

Response：**The SARIMA model is developed on the basis of ARIMA model. If the original series has obvious time fluctuation trend and seasonal characteristics, SARIMA model can be considered.**

17) Line 97: This sentence does not make sense, please revise for grammar.

Response：**It has been corrected to: After the sequence is stabilized by seasonal and non seasonal difference.**

18)Line 98: The order of the model is determined by the autocorrelation function and the AIC information criterion?

Response：**The approximate order of the model is determined according to the autocorrelation function. Then the AIC information criterion is used to determine the optimal parameter combination of the model**

19) Equation 2: The authors could consider removing this equation and simply reference Burnham and Anderson 2002; in fact, there is no reference to Burnham and Anderson 2002 and there should be for AIC.

Response：**It has been corrected in the manuscript.**

Results:

Generally, it is difficult to understand and interpret the results because of the insufficient methods section.

20) Line 130: Do the authors mean from 0-6 on a 24hr basis? If so how does it reach a minimum at 6pm? And if it increases slowly from 6 am, then at 14:00 that would be 2pm which is before 6pm. It appears the time is incorrectly reported, please revise.

Response：**There should be no “PM” here. It’s my negligence. It has been corrected in the manuscript.**

21) Line 127- 136: The authors should reconsider describing the trends presented in the graphs, and providing results that are not easily communicated or presented in the graphs.

Response：**We have added some content to the manuscript.**

22) Line 139: What are production and life rules, and what evidence supports this conclusion?

Response：**It's the certain mode of production and living habits.** **It's been corrected in the manuscript.**

23) Line 143: What are above fires?

Response： **the proportion of major fires with more than 3 deaths is high，**

24) Line 141-148: This seems like a “common sense” conclusion but there is no evidence presented for this. I do not think the authors have spent time analyzing why the patterns they present occurred, just seems like conventional wisdom was deployed.

Response：**Some modifications have been made in the manuscript.**

25) Line 158-165: Again these conclusions just do not seem like they are steeped in any kind of analyses.

Response：**Through literature review, the proportion of fire caused by electrical causes is as high as 40% in the whole country. In winter and spring, people use fire, electricity and gas intensively, and the fire causing factors increase. In addition, according to the statistics of the China Fire Service, the Chinese traditional festival Spring Festival is generally from January to February. In the past 15 years, the proportion of fires during the Spring Festival (6 days from New Year's Eve to the fifth day) accounts for 30% - 45% of that month's fires. There are also many fire accidents caused by fireworks during the festival.**

Once again, thank you very much for your constructive comments and suggestions which would help us both in English and in depth to improve the quality of the paper.

Kind regards.

Shuqi Ma

E-mail: shuqi_10@163.com

Corresponding author : Qianyi LIU, Yudong ZHANG

E-mail address: [lqy202103@163.com](mailto:lqy202103@163.com); zhangyudong@tsinghua.edu.cn
